# Comparing half-dose photodynamic therapy with high-density subthreshold micropulse laser treatment in patients with chronic central serous chorioretinopathy (the PLACE trial): study protocol for a randomized controlled trial

**DOI:** 10.1186/s13063-015-0939-z

**Published:** 2015-09-21

**Authors:** Myrte B. Breukink, Susan M. Downes, Giuseppe Querques, Elon H. C. van Dijk, Anneke I. den Hollander, Rocio Blanco-Garavito, Jan E. E. Keunen, Eric H. Souied, Robert E. MacLaren, Carel B. Hoyng, Sascha Fauser, Camiel J. F. Boon

**Affiliations:** Department of Ophthalmology, Radboud University Medical Center, Philips van Leydenlaan 15, 6525 EX Nijmegen, The Netherlands; Oxford Eye Hospital, John Radcliffe Hospital, West Wing Headington, Oxford, OX3 9DU UK; Department of Ophthalmology, University Paris Est Creteil, Center Hospitalier Intercommunal de Creteil, 40 avenue du Verdun, 94000 Creteil, France; Department of Ophthalmology, Leiden University Medical Center, Albinusdreef 2, 2333 ZA Leiden, The Netherlands; Department of Ophthalmology and Department of Human Genetics, Radboud University Medical Center, Philips van Leydenlaan 15, 6525 EX Nijmegen, The Netherlands; Department of Ophthalmology, University Hospital of Cologne, Kerpener Strasse 62, 50924 Cologne, Germany

**Keywords:** chronic central serous chorioretinopathy, half-dose photodynamic therapy, high-density subthreshold micropulse laser, prospective, randomized controlled, multicenter, trial, verteporfin

## Abstract

**Background:**

Chronic central serous chorioretinopathy (cCSC) is an eye disease characterized by an accumulation of serous fluid under the retina. It is postulated that this fluid accumulation results from hyperpermeability and swelling of the choroid, the underlying vascular tissue of the eye, causing a dysfunction of the retinal pigment epithelium. This fluid accumulation causes neuroretinal detachment. A prolonged neuroretinal detachment in the macula can lead to permanent vision loss. Therefore, treatment is aimed primarily at achieving resolution of subretinal fluid, preferably within the first 4 months after diagnosis of the disease. A broad spectrum of treatment modalities has been investigated in cCSC, but no consensus exists on the optimal treatment of cCSC. Currently, photodynamic therapy (PDT) and high-density subthreshold micropulse laser treatment (HSML) are among the most frequently cited treatments in obtaining successful neuroretinal reattachment.

**Methods/Design:**

This is a randomized, controlled, open-label, multicenter trial comparing the efficacy of half-dose PDT to HSML in treating patients with cCSC. A total of 156 patients will be recruited, 78 patients in each treatment arm, with a maximum follow-up duration of 8 months after the first treatment. A complete ophthalmological examination with vision-related quality of life (NEI VFQ-25) and stress questionnaires, will be performed at baseline, 6 to 8 weeks after the first treatment, 6 to 8 weeks after a second treatment (if necessary), and at the final follow-up visit at 7 to 8 months after the first treatment. Treatment visits will be scheduled within 3 weeks after the baseline visit, and within 3 weeks after the first control visit, if a second treatment is required.

**Discussion:**

Both half-dose PDT and HSML may be effective treatments in cCSC, but because of the lack of prospective randomized controlled trials, which treatment should be the first choice remains unclear. The aim of this study is to compare the efficacy of half-dose PDT to HSML. The primary endpoint to evaluate efficacy will be a complete absence of subretinal fluid on optical coherence tomography after treatment. Secondary functional endpoints include change in Early Treatment Diabetic Retinopathy Study (ETDRS) best-corrected visual acuity, retinal sensitivity on microperimetry, and NEI VFQ-25 questionnaire of visual functioning.

Registration number Institutional Review Board (CMO Arnhem-Nijmegen, the Netherlands): 2013/203 NL nr.: 41266.091.13

**Trial registration:**

ClinicalTrials.gov identifier: NCT01797861. Date of registration: 21 February 2013.

## Background

### Chronic central serous chorioretinopathy

Central serous chorioretinopathy (CSC) is a relatively common early-onset eye disease, characterized by an accumulation of leaked serous fluid under the retina, causing a detachment of the neuroretina. This subretinal fluid (SRF) leakage results from dysfunction of the retinal pigment epithelium (RPE), and the presence of choroidal congestion and thickening and hyperpermeability of the choroid implies an important role for choroidal abnormalities as an underlying cause for RPE dysfunction and SRF leakage in CSC [1–4].

**Fig. 1:**
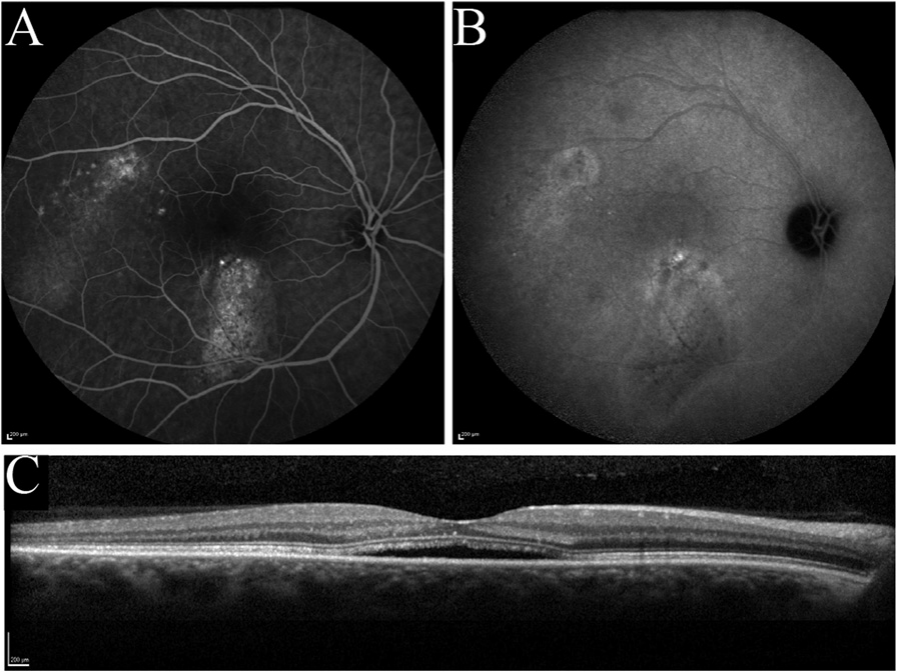
Multimodal imaging in chronic central serous chorioretinopathy. Examples of fluorescein angiography (FA), indocyanine green (ICG) angiography and spectral-domain optical coherence tomography (SD-OCT) in chronic central serous chorioretinopathy (cCSC). (**a-c**) Right eye of a patient with cCSC with more widespread leakage on FA (**a**) corresponding with hyperfluorescent areas on ICG angiography (**b**) and SRF on SD-OCT (**c**)

Two main subtypes of CSC are generally distinguished: acute and chronic CSC (cCSC) [1–7]. Patients with acute CSC present with a sudden and marked central vision loss because of SRF leakage in the macula; this is due to a focal leak in the RPE that is visible on fluorescein angiography. Acute CSC generally has a favorable prognosis because the SRF often disappears spontaneously within 2 to 3 months, with either complete or almost complete recovery of vision. In contrast, cCSC is typically not self-limiting, and SRF persists for more than 3 months. Also, cCSC patients present at an older age, with a disease onset that is generally experienced as less sudden, and bilaterality in chronic CSC is common [8]. A history of acute CSC and/or an episode of acute vision loss compatible with acute CSC is present only rarely in chronic CSC patients [2, 8], which also points to a distinction between acute and chronic CSC. Patients with the cCSC phenotype have more diffuse multifocal leakage on fluorescein and indocyanine green (ICG) angiography, as well as irregularly distributed widespread RPE changes associated with varying degrees of more indistinct leakage on angiography (Fig. 1). Persistent serous neuroretinal detachments can cause progressive and irreversible photoreceptor damage, resulting in a poorer visual prognosis of cCSC as compared to acute CSC [2, 9, 10]. The etiology of CSC is largely unknown, but the use of corticosteroids is a risk factor, and possibly elevated cortisol levels, stress, “type A” personality, and pregnancy are also possible risk factors [1, 11–14]. The incidence of CSC is approximately six times higher in men than in women [1], although this male-to-female proportion seems to be less pronounced in cCSC and steroid-associated CSC. Recently, single-nucleotide polymorphisms in the *Complement Factor H* and *ARMS2* have been found to be associated with cCSC [15, 16].

A prolonged neuroretinal detachment in the macula leads to progressive and permanent central visual loss due to photoreceptor atrophy. In addition, Piccolino et al. described the occurrence of not only SRF, but also of intraretinal fluid accumulations, posterior cystoid degeneration, and prolonged neuroretinal detachments in severe cCSC cases [17]. Nicolo et al. have shown that this posterior cystoid degeneration is associated with a poorer response to photodynamic therapy (PDT) [17, 18]. A loss of visual acuity with image distortion and a loss of color and contrast vision may have a significant impact on a patient’s personal and professional life. Early diagnosis and treatment is important to try to improve the visual outcome and quality of life, as long-term follow-up studies have shown that the natural course of cCSC often results in permanent visual loss [3, 9, 10, 19–25]. Therefore, several treatment options have emerged in an attempt to accelerate the resolution of SRF accumulation and to improve the visual outcome in patients with cCSC. Treatment within 4 months after the onset of the disease has been advocated by several authors, based on the observation that permanent visual loss may result from prolonged duration of disease [2, 3]. Photoreceptor atrophy in the fovea may occur, even after successful reattachment of the retina, after a duration of symptoms of approximately 4 months [9]. To date there is no international consensus on the optimal treatment protocol of cCSC.

### Photodynamic therapy in chronic central serous chorioretinopathy

A number of retrospective studies suggests that in 70 to 100 % of CSC patients, treatment with PDT, using the photosensitizing drug verteporfin (Visudyne™), is effective in reducing SRF, with an improvement of retinal anatomy, visual acuity [18, 26–30], and retinal sensitivity [31–35]. PDT treatment was developed originally as treatment for neovascular age-related macular degeneration, on which there are extensive data available [36, 37]. There are several other retinal diseases for which PDT with verteporfin is successfully used as an off-label treatment, such as choroidal hemangioma and polypoidal choroidal vasculopathy [37].

PDT with Visudyne™ (verteporfin for injection) is a two-stage procedure that first requires the intravenous administration of verteporfin, followed by the administration of non-thermal red light into the affected eye. Verteporfin is transported in the plasma primarily by lipoproteins. Once verteporfin is activated by light in the presence of oxygen, highly reactive, short-lived singlet oxygen and reactive oxygen radicals are generated. Verteporfin appears to accumulate preferentially in abnormal neovascularization (which is not present in cCSC) but also in the choroidal vasculature. The latter mechanism is of special interest in the treatment of cCSC because CSC primarily affects the choroidal circulation, resulting in multifocal areas of choroidal vascular hyperpermeability that may finally result in the accumulation of SRF. The therapeutic effect of PDT in cCSC is thought to result from short-term choriocapillaris hypoperfusion and long-term choroidal vascular remodeling, leading to reduction in choroidal congestion, vascular hyperpermeability, and extravascular leakage [38–40].

As mentioned previously, there is no international consensus on the optimal treatment protocol of cCSC. Nevertheless, PDT has emerged as the treatment of choice in many centers worldwide, based on the high rate of anatomic success, the increase of visual acuity, improvement in retinal sensitivity, and an excellent safety profile reported in many retrospective studies [3, 41–43]. The PDT strategies that are generally used are either with half the dose of verteporfin and full fluency (energy) of laser treatment, half the fluency level and the full dose of verteporfin, or half the treatment time using the full dose of verteporfin and full fluency, as compared to the original protocol that was used for neovascular age-related macular degeneration. These PDT strategies that use either half-dose of half-fluency treatment have been developed because a combination of the dosage and fluency that was originally used for the treatment of neovascular age-related macular degeneration showed a potentially higher risk of developing choroidal ischemia and retinal atrophic changes [36, 44–46]. The half-dose or half-fluency PDT strategies, however, have been shown to be safe and effective in relatively large retrospective studies and in one noncontrolled, nonrandomized prospective study by Chan et al. in cCSC patients with sufficient follow-up periods [3, 27, 29, 41].

Therefore, tailoring the therapy to obtain the maximal treatment effect with minimal toxicity is essential in treating patients with CSC. By reducing the dose of verteporfin, studies have demonstrated that the potential retinal damage caused by PDT can be minimized while the photodynamic effects in inducing choroidal vasculature changes required for treating CSC remain sufficient [2, 3, 18, 26–30, 41, 47]. None of the patients treated with this half-dose PDT protocol experienced any systemic adverse event (AE) associated with verteporfin infusion [27]. In several relatively large retrospective studies on half-dose PDT in CSC, none of the patients had any subjective or objective drop in vision immediately after PDT or at subsequent follow-up visits [18, 26–30]. This “safety-enhanced” protocol with half-dose verteporfin appeared to be one of the safest and effective treatment options in patients with active cCSC [23, 41]. In conclusion, a relatively large body of well-documented retrospective studies indicates that half-dose PDT is able to yield positive functional and anatomic outcomes while at the same time reducing the potential AEs associated with conventional PDT with full-dose verteporfin.

### High-density subthreshold micropulse laser therapy as an alternative treatment in chronic central serous chorioretinopathy

There are several retrospective studies that indicate that high-density subthreshold micropulse laser (HSML) therapy may be effective in 41 to 58 % of CSC patients [18, 48]. HSML treatment using a 810 nm wavelength is an established treatment option for a broad range of retinal diseases [49]. In this treatment, no photosensitizing drug is needed. This relatively new laser treatment modality may prevent damage to the neural retina that occurs in conventional (non-PDT) laser techniques by raising the temperature of the RPE below the protein-denaturation-threshold so that the thermal wave that reaches the neural retina is insufficient to cause either damage or a clinically visible end-point (opacified retina). It is different from subthreshold continuous wave in that more energy can be delivered to the RPE without neuroretinal damage using multiple short pulses. In contrast, in the continuous wave mode of conventional laser therapy, the laser energy is delivered with a single pulse with a duration of exposure of 0.1 to 0.5 s, most of the energy is absorbed by the RPE and the heat energy is transferred to the neurosensory retina leading to transient retinal swelling (visible end-point). As a result, conventional laser application in the macula may cause damage to the neuroretina and RPE, leading to central scotomas and possibly loss of visual acuity. Previous studies have shown that conventional laser treatment in CSC, in contrast to HSML treatment and half-dose PDT treatment, does not improve visual acuity, may cause photoreceptor damage, and may induce choroidal neovascularization [3]. Recently, studies using a relatively new 577 nm wavelength micropulse laser in subthreshold mode have also shown possible efficacy in the treatment of cCSC [50, 51].

### Outline of proposed clinical trial

The PLACE study is a superiority study because retrospective studies suggest that the rate of anatomical and functional success of PDT treatment might be higher than the success of HSML treatment. Therefore, a half-dose PDT treatment arm is challenged against a treatment arm of HSML treatment.

In this study, we want to define treatment success not only on the basis of structural parameters (anatomic success, for example, the absence of SRF after treatment), but also based on functional vision-related endpoints, which are most important from a patient’s perspective. These functional vision-related endpoints will include best-corrected visual acuity (BCVA), retinal sensitivity on microperimetry and score on a validated visual function questionnaire (the NEI-VFQ-25 questionnaire) http://www.psy.cmu.edu/~scohen/ [52].

With the results of this study, we hope to establish a strong scientific foundation for further research on the optimal treatment of patients with cCSC to improve the visual outcome and quality of life of this relatively frequently occurring eye disease.

### Objectives

#### Primary objective

The primary objective of this study is to investigate whether treatment of cCSC patients with macular SRF on optical coherence tomography (OCT) with half-dose PDT results in more eyes with an absence of SRF on the OCT as compared to HSML treatment.

#### Secondary objectives

The secondary objectives are to investigate the clinical outcome comparing half-dose PDT treatment with HSML treatment in patients with SRF due to active leakage in cCSC, based on evaluation of BCVA; the retinal sensitivity on microperimetry; and the subjective success score on the NEI-VFQ-25 questionnaire.

## Methods/Design

### Summary of trial design

**Fig. 2:**
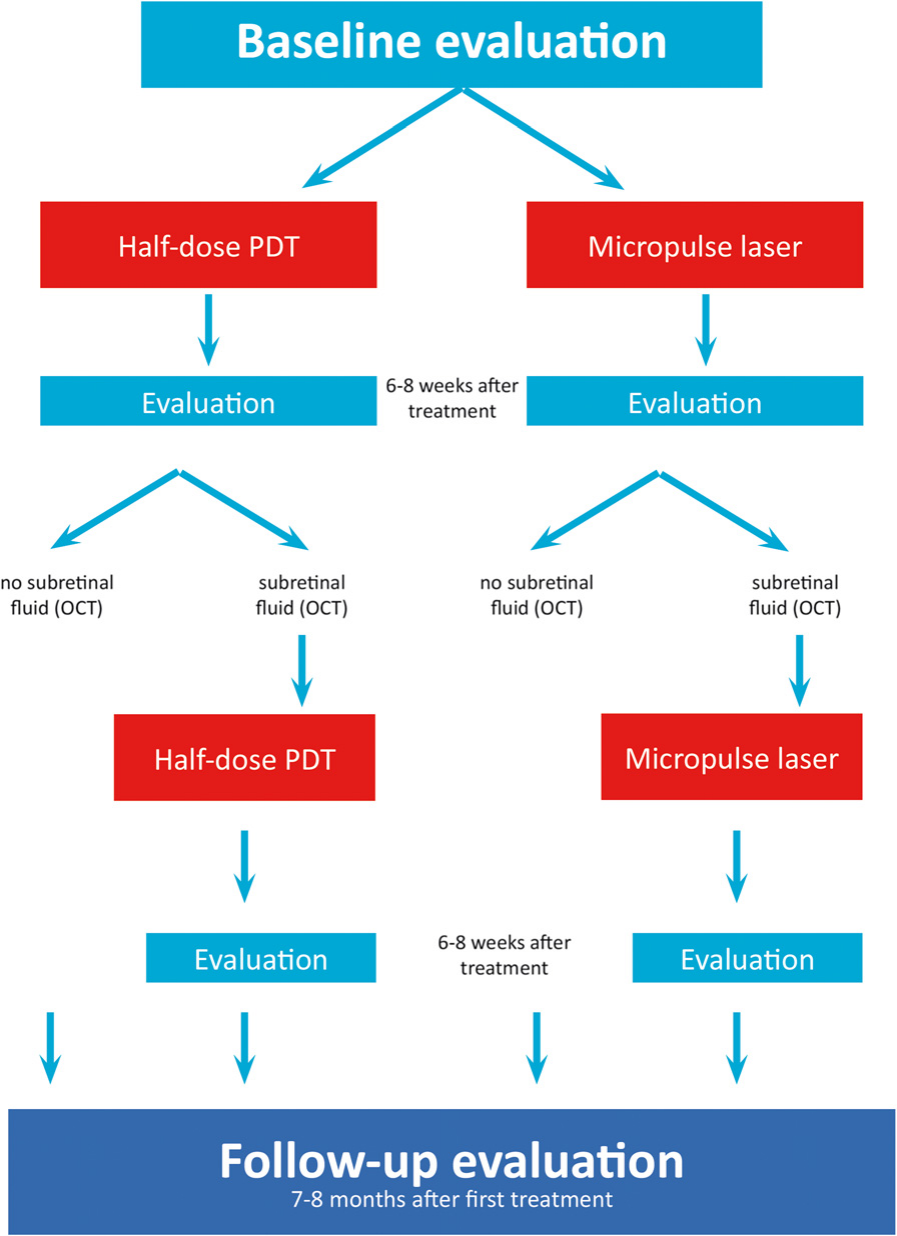
Study flow chart

This study is a multicenter, prospective, randomized, controlled, open-label study that will compare the efficacy and safety of two treatments in patients with cCSC. The first group of patients will receive half-dose PDT treatment. The second group of patients will receive 810 nm HSML treatment. Each patient will receive at least one treatment but may be eligible to receive a second treatment during follow-up (Fig. 2), which will be the same type of treatment as the first treatment: either half-dose PDT treatment or HSML treatment.

Potential eligible patients will be identified in one of five specialist ophthalmology trial sites, each led by one of the principal investigators (PIs).

Fundus photographs, fluorescein angiograms, ICG angiograms and OCT images collected at the screening visit will be sent to a central reading center (CRC). The CRC will review these images to confirm subject eligibility based on the characteristics specified in the inclusion and exclusion criteria. Once eligibility has been confirmed by the CRC, all other inclusion and exclusion criteria have been met at the baseline visit, and informed consent has been obtained, patients will be enrolled in the trial.

There are nine examinations that will be performed at the baseline assessment, 6 to 8 weeks after treatment (at evaluation visit 1, and if a second treatment is required, at evaluation visit 2), and at the final visit (7 to 8 months after start of the treatment). The six *anatomical* assessments include ophthalmoscopy, fundus photography, OCT, autofluorescence imaging, fluorescein angiography, and ICG angiography. The three *functional* assessments include visual acuity measurement, microperimetry, and a questionnaire on vision-related functioning.

Enrolled patients will be randomized at a 1:1 ratio to receive either half-dose PDT treatment or HSML treatment.

The total number of visits per patient is five (in case of one required treatment) or seven (in case of two required treatments). The duration of participant participation within the study is 7 to 8 months (Fig. 2).

An overview of the assessments in the trial are as follows:

1. Ophthalmoscopy
  This examination will be performed by one of the ophthalmologists to confirm the diagnosis. The pupils will have to be dilated with 1.0 % tropicamide and 2.5 % phenylephrine for ophthalmoscopy to be performed.
2. Fundus photography
  The fundus photography will be taken with a Topcon TRC-50 series fundus camera. The photographs will be taken with a 50°-field centered on the area of the macula.
3. Optical coherence tomography (OCT) imaging
  Patients will be examined by noninvasive OCT imaging. OCT shows the different layers of the retina and is the imaging modality of choice to visualize subretinal and intraretinal fluid, for instance in cCSC. This examination is performed during mydriasis (dilated pupils). OCT imaging will be carried out with a Spectralis HRA + OCT (Heidelberg Engineering, Heidelberg, Germany).
4. Fundus autofluorescence imaging
  Fundus autofluorescence is a noninvasive imaging technique that is able to register changes of autofluorescence intensity in the retina, for instance due to accumulation of lipofuscin in the RPE. Fundus autofluorescence imaging will be carried out with a Spectralis HRA + OCT (Heidelberg Engineering, Heidelberg, Germany).
5. Fluorescein and indocyanine green (ICG) angiography
  To perform fluorescein and ICG angiography, intravenous injection of fluorescein and ICG is required. During the angiography procedure, both (fluorescein and ICG) dyes may be injected at the same time or separately. Fluorescein reveals the retinal vasculature and may show areas of fluid leakage through the RPE, whereas the ICG dye images the choroidal vasculature. The visualization of both the retinal vasculature and RPE permeability (by fluorescein angiography) and choroidal vasculature (by ICG angiography) is essential to image the areas of abnormal anatomy and leakage that may guide treatment. Images of the study eye are taken at set times: 0 to 1 minute (several images covering the arterial and venous filling phases), 3 minutes, 6 minutes, 10 minutes, 15 minutes, and 20 minutes. Images of the nonstudy eye will be taken at 1 minute, 6 minutes, and 20 minutes. All images will be acquired with Spectralis HRA + OCT (Heidelberg Engineering, Heidelberg, Germany).
6. Best-corrected visual acuity (BCVA)
  BCVA will be assessed for both eyes at all evaluation visits. To measure BCVA, early treatment diabetic retinopathy study (ETDRS) visual acuity testing charts will be used at a distance of 4 meters.
7. Microperimetry
  All patients will be examined by noninvasive microperimetry. This technique is able to measure retinal sensitivity to light, and follow-up pre- and post-treatment changes in retinal sensitivity at predetermined loci in the macula. By using a reliable eye tracking system, this microperimetry system is able to exactly locate the areas of retina that have been tested previously, in order to ensure testing of identically the same area at follow-up. Microperimetry according to a standard protocol takes 5 to 8 minutes for each eye and will be performed with non-dilated pupils.
8. Questionnaires
  Each participant will be asked to complete a quality-of-life questionnaire based on the National Eye Institute Visual Function Questionnaire (NEI-VFQ-25) [52], and the Cohen Stress Questionnaire [53] [http://www.centervue.com/product.php?id=639]. NEI-VFQ-25 is a reliable and validated 25-item version of the 51-item National Eye Institute Visual Function Questionnaire [54]. The questionnaire is especially useful in settings such as clinical trials, where interview length is an important consideration. The Cohen Stress Questionnaire is a validated questionnaire indicating the stress level patients have been exposed to during the month before disease onset.

There will be standard operating procedures (SOPs) available to all investigators involved in the trial as well as in the trial master file for each of the described examinations.

### Primary and secondary endpoints

#### Primary endpoint

The primary endpoint of this study is to assess if there is a difference between the efficacy of half-dose PDT treatment versus the HSML treatment in patients with cCSC. The assessment of this efficacy will be based on the anatomical effect on OCT: absence of SRF versus persistent SRF, 6 to 8 weeks after treatment. After all, the absence or presence of fluid under the retina on the OCT scan is a direct reflection of the activity of the disease in these patients.

#### Secondary endpoints

For secondary endpoints, we will mainly look at three parameters that reflect the patient’s vision-related functioning. These three parameters are a standardized measurement of BCVA according to the ETDRS standards, a standardized measurement of sensitivity of the macula with microperimetry, and a standardized assessment of the patient’s vision-related quality of life using a validated questionnaire, the NEI-VFQ-25.

The secondary endpoints that will be assessed as a reflection of functional improvement after treatment include the following:

1. Number of second treatments needed in each treatment arm.
2. Mean change from baseline in ETDRS BCVA in the study eye at 6 to 8 weeks after treatment visit 1 and at 7 to 8 months after treatment visit 1, among the two treatment modalities.
3. Mean change from evaluation visit 1 in ETDRS BCVA in the study eye at final evaluation (7 to 8 months after treatment visit 1), among those who required one treatment and those who required a second treatment, and among the two treatment modalities overall.
4. Mean change from baseline in retinal sensitivity on microperimetry in the study eye at 6 to 8 weeks after treatment visit 1 and at 7 to 8 months after treatment visit 1 among the two treatment modalities.
5. Mean change from baseline in the NEI-VFQ-25 questionnaire at 6 to 8 weeks after treatment visit 1 and at 7 to 8 months after treatment visit 1 among the two treatment modalities.
6. An absence of SRF on evaluation with OCT scanning as compared to HSML treatment at 7 to 8 months follow-up after successful treatment (after treatment visit 1; “success” defined as an absence of SRF on OCT at 6 to 8 weeks after treatment).

### Trial participants

#### Overall description of the trial participants

This study will enroll subjects with cCSC with active leakage of fluid under the retina as evidenced on OCT scanning and further supported by findings on fluorescein angiography and ICG angiography, in at least one eye. If both eyes are eligible, then the eye with the longer duration of disease will be used as the study eye, except in cases where the disease is present for more than 18 months. In the latter case, which is an exclusion criterion, the other eye will be eligible for inclusion if the disease has been active for less than 18 months. If the nonstudy eye also has active disease, the choice to treat and the type of treatment in this eye may be chosen freely at the discretion of the responsible ophthalmologist.

Before enrolment, each subject must meet all of the following inclusion criteria and none of the exclusion criteria and agree to comply with the study requirements, including completion of all of the study visits.

#### Inclusion criteria

The inclusion criteria are as follows:

1. Male and female patients ≥18 years of age who are able to give written informed consent.
2. Active cCSC.
3. Subjective visual loss >6 weeks, interpreted as onset of active disease.
4. SRF that includes the fovea on OCT scanning at baseline examination. *Note*: SRF does *not* have to include fovea on OCT to be eligible for treatment at control visit 1 as long as there is persistent SRF in the macula, which is interpreted as persistently active disease.
5. Hyperfluorescent areas on ICG angiography.
6. ≥ 1 ill-defined hyperfluorescent leakage areas on fluorescein angiography with RPE window defect(s) that are compatible with cCSC.

#### Exclusion criteria

The participant may not enter the study if any of the following apply:

1. Any previous treatments for active CSC in the study eye.
2. Current treatment with corticosteroids (topical or systemic), corticosteroid use within 3 months before the possible start of trial treatment, or anticipated start of corticosteroid treatment within the first 7 to 8 months from the start of the trial period.
3. Evidence of other diagnosis that can explain serous SRF or visual loss.
4. BCVA <20/200 (Snellen equivalent).
5. Profound chorioretinal atrophy in central macular area on ophthalmoscopy and OCT.
6. Myopia >6D.
7. Visual loss and/or serous detachment on OCT <6 weeks.
8. Continuous and/or progressive visual loss >18 months or serous detachment on OCT >18 months.
9. No hyperfluorescence on ICG angiography.
10. Intraretinal edema on OCT.
11. Contraindications (relative) for PDT treatment (pregnancy, porphyria, severely disturbed liver function). Pregnancy will not be routinely tested in female patients, but the possibility of pregnancy will be discussed during eligibility screening.
12. Contraindications (relative) for fluorescein angiography or ICG angiography (known allergies especially against shellfish, previous reactions).
13. Soft drusen in treated eye or fellow eye, signs of choroidal neovascularization on ophthalmoscopy and/or fluorescein angiography/ICG angiography.

### Study procedures

#### Screening and eligibility assessment

##### Identification of potential participants

Potential participants with cCSC will be identified in the participating trial sites, after being referred to the department by the general practitioner (GP) or referring ophthalmologists from other hospitals. Before screening, a visual acuity measurement, dilated ophthalmoscopy, fundus photography, OCT of the retina and choroid, autofluorescence imaging, and fluorescein and ICG angiography will already have been performed in most patients as part of standard clinical care. These examinations constitute most of the baseline examinations and therefore do not have to be repeated if screening and randomization is performed within 2 weeks after these examinations. Screening and baseline examinations/enrolment are performed on the same day if possible. The maximum duration allowed between screening and randomization is 2 weeks.

In addition to the examinations mentioned previously, the following information will be collected from patients who have been consented, at the baseline assessment; at control visit 1 and, if applicable, at control visit 2; and at the follow-up visit at 7 to 8 months after treatment visit 1:

1. Demographic details - the date of birth, gender, race, smoking and drinking habits will be recorded on case report forms (CRFs).
2. Medical history - details of any history of disease or surgical interventions will be recorded on CRFs.
3. Concomitant medication - all over-the-counter or prescribed medication, vitamins, and/or herbal supplements will be recorded on CRFs.

#### Informed consent

The study will be discussed with the subject. The patient information sheet will be given to the patient at the screening visit, and the patient will be asked to contact us if he/she is willing to take part. A subject wishing to participate must give written informed consent prior to any study-related procedures or change in treatment. The participant must personally sign and date the latest approved version of the informed consent form before any study-specific procedures are performed. Written and verbal versions of the participant information and informed consent will be presented to the participants, detailing the exact nature of the study, the implications and constraints of the protocol, the known side effects, and any risks involved in taking part. It will be clearly stated that the participant is free to withdraw from the study at any time for any reason without prejudice to future care, and with no obligation to give the reason for withdrawal.

The participant will be allowed as much time as wished to consider the information, and the opportunity to question the investigator, their GP or other independent parties to decide whether they will participate in the study. Written informed consent will then be obtained by means of the participant’s dated signature and dated signature of the person who presented and obtained the informed consent. The person who obtained the consent must be suitably qualified and experienced and have been authorized to do so by the chief/principal investigator. A copy of the signed informed consent will be given to the participants. The original signed form will be retained at the study site, and an additional copy will remain in the patient notes.

#### Randomization

Subject numbers will be assigned sequentially as each subject enters the study.

The subjects will be assigned to a study treatment by a web-based random numbers generator using block randomization without minimization. This application will be specially designed for this study by the Department of Epidemiology, Biostatistics and Health Technology Assessment of the Radboud University Nijmegen Medical Center (Nijmegen, the Netherlands). Randomization will be performed at the same visit as the baseline visit. The randomization schedule is designed by a statistician, and the randomization codes are kept in the CRF and in the digital database of the Clinical Research Center Nijmegen (www.CRCN.nl).

#### Interventions

##### Half-dose PDT treatment

**Fig. 3:**
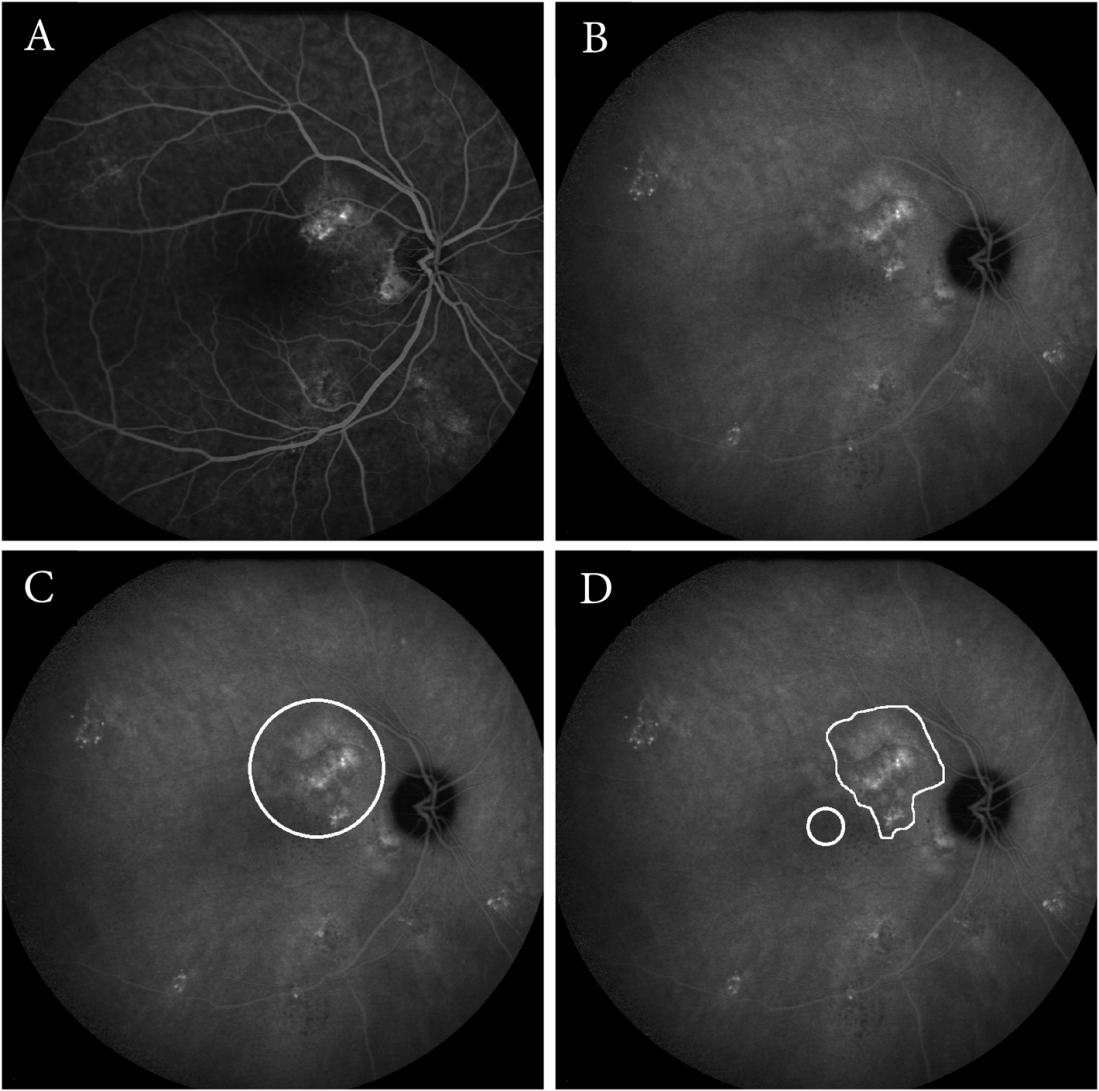
Examples of areas treated in photodynamic therapy and micropulse laser treatment. Examples of imaging features on fluorescein angiography (FA) and indocyanine green angiography (ICG) angiography in chronic central serous chorioretinopathy (cCSC), and the corresponding treatment areas for photodynamic therapy (PDT) and high-density subthreshold micropulse laser treatment (HSML). **a-b** FA of the right eye of a patient showing hyperfluorescent “hot spots,” indicating leakage inferior of the fovea (**a**). On ICG angiography, an area of hyperfluorescence, which corresponds to the hyperfluorescent area on the FA, is seen (**b**). **c-f** An example of a PDT spot (white circle) overlapping the hyperfluorescent area on the ICG angiography plus 1 mm as described in the protocol (**c**). HSML treatment scheme that would apply to the same eye, in which only the central foveal area is excluded for treatment (white circle). The hyperfluorescent area on the ICGA is treated with numerous, nonoverlapping adjacent laser spots (white area) (**d**)

For this intervention, the patients need dilated pupils (dilation is achieved with 1.0 % tropicamide and 2.5 % phenylephrine). All patients will get an intravenous infusion of 3 mg/m^2^ verteporfin (Visudyne ™) (half-dose) over a period of 10 minutes. At exactly 15 minutes after the start of the infusion, an anesthetic eye drop is given (oxybuprocaine 0.4 % or equivalent), a contact lens (a Volk™ PDT lens) is positioned on the affected eye, and the aiming beam of the laser is focused on the treatment area. The magnification factor is taken into account in the settings of the PDT machine. The area of treatment is chosen, with the area of the aiming beam corresponding to the area of the subsequent laser spot area. The area that has to be treated is determined based on the hyperfluorescent area(s) on midphase (approximately 10 minutes) ICG angiography that correspond(s) to the SRF accumulation in the macula on the OCT scan and hyperfluorescent “hot spots” on the midphase (approximately 3 minutes) fluorescein angiogram. The spot size will be defined based on the diameter of the hyperfluorescent area on the ICG angiography plus 1 mm (Fig. 3). The edge of treatment spot has to be *at least 200 μm away from the optic disc rim*. The PDT treatment is performed with standard 50 J/cm^2^ fluency, a PDT laser wavelength of 689 nm, and a standard treatment duration of 83 seconds. Care must be taken to treat at *exactly* 15 minutes after the start of the infusion, to maximize the localization of the effect of treatment to the choroid and minimize possible damage to the adjacent retinal structures. The PDT treatment must take place at least 45 minutes after ICG angiography has been performed.

##### HSML treatment

For this intervention, the patients need dilated pupils (dilation achieved with 1.0 % tropicamide and 2.5 % phenylephrine). An anesthetic eye drop is given (oxybuprocaine 0.4 % or equivalent), and a contact glass (for instance a Volk™ area centralis lens) is positioned on the affected eye. HSML treatment with an 810 nm diode laser will be performed of the areas identified on midphase ICG angiography. Multiple confluent, adjacent (nonoverlapping) laser spots will be applied, covering the leakage area on midphase ICG angiography. The number of spots and number of zones treated depends on the extent of the leakage area(s) on midphase ICG. The area that has to be treated is determined based on those hyperfluorescent area(s) on midphase (approximately 10 minutes) ICG angiography that correspond(s) to SRF accumulation in the macula on the OCT scan and hyperfluorescent “hot spots” on the midphase (3 minutes) fluorescein angiogram (Fig. 3). The treatment will consist of small adjacent laser spots covering the designated area *keeping a distance of 500 μm from the foveal center (corresponding to a laser-free circular zone of 1000 μm diameter centered on the fovea)*.

The following *HSML treatment settings* will be used: a power of 1800 mW, a duty cycle of 5 %, frequency of 500 Hz, exposure time of 0.2 s per spot, a spot size of 125 μm, and a minimal distance of the spot from the fovea of 500 μm [48, 55]. 

Subthreshold treatment is desired, meaning that no visible reaction due to laser treatment has to be seen in the retina. In virtually all patients, a power of 1800 mW will not produce a visible discoloration of the retina after application of a laser spot with the aforementioned settings. If retinal discoloration is seen at a power of 1800 mW (corresponding to suprathreshold treatment), for instance in patients with darkly pigmented fundi, the power will be reduced with steps of 300 mW until there is no visible reaction. The first laser “test” spot will always be applied just outside the macular area.

#### Retreatment criteria and considerations

At evaluation visit 1, at 6 to 8 weeks after treatment, an OCT scan of the retina will be performed, among other imaging examinations. If there still is SRF present in the macular area, a second treatment will be performed according to the protocol within 3 weeks after evaluation visit 1.

This second treatment (either half-dose PDT or HSML treatment) will again be guided by the hyperfluorescent area(s) on the ICG angiography that correspond(s) to SRF accumulation in the macula on the OCT scan and the hyperfluorescent “hot spots” on the midphase (approximately 3 minutes) fluorescein angiogram.

In cases where there is no more SRF under the fovea but there is persistent fluid within the macular area encircled by the optic disc and temporal retinal vascular arcade, retreatment will be performed. The rationale behind this second treatment is that the persistent fluid may be interpreted as an incomplete treatment response because SRF accumulation indicates ongoing disease activity due to choroidal vascular hyperpermeability and fluid leakage through the RPE.

Note that if a patient did not require retreatment at evaluation visit 1 (=6-8 weeks after treatment visit 1) according to the protocol but returns with visual symptoms in the period between evaluation visit 1 and the follow-up visit at 7 to 8 months, a regular clinical examination should be performed that includes at least a measurement of visual acuity, ophthalmoscopy, and OCT scan to determine whether SRF has reoccurred. If there is evidence of recurrence of SRF on OCT (and therefore disease activity), these findings should be noted in the CRF. In these cases, additional evaluations compatible with evaluation visit 2 should be performed, and the patient should be planned for treatment visit 2 (max. 2 to 3 weeks after evaluation visit 2). Further evaluation will adhere to the retreatment criteria described above.

However, no treatment is allowed if the patient has already been treated twice (either with two half-dose PDT treatments or two HSML treatments) according to the trial protocol between evaluation visit 1/2 and follow-up visit (7 to 8 months after treatment visit 1). There are no data on the usefulness of more than two half-dose PDT treatments in the same patient with persistent cCSC. Some authors argue that more than two PDT treatments in the same eye may increase the risk of complications such as choroidal ischemia and the formation of choroidal neovascularization. In the case of HSML treatment, there also are no reports to suggest that more than two treatments may be useful. That is why a maximum amount of two of the same treatments is allowed within the trial period. After completion of the trial (at follow-up visit, 7 to 8 months after treatment visit 1), treatment may be considered and the treatment modality may be chosen at the discretion of the treating ophthalmologist.

#### Definition of end of trial

The end of trial is the date on which the last included participant has received the last follow-up visit (7 to 8 months after treatment 1).

#### Discontinuation and withdrawal of participants from study treatment

Each participant has the right to withdraw from the study at any time. In addition, the investigator may discontinue a participant from the study at any time if the investigator considers it necessary for any reason including the following:

1. Ineligibility (either arising during the study or retrospective having been overlooked at screening).
2. Significant protocol deviation.
3. Significant non-compliance with study requirements.
4. An AE that requires discontinuation of the study medication or results in inability to continue to comply with study procedures.
5. Consent withdrawn.
6. Lost to follow-up.
7. Pregnancy before evaluation visit 1 or 2 (which is a relative contraindication for angiography). Pregnancy will not be routinely tested in female patients, but the possibility of pregnancy will be discussed during eligibility screening.

Patients suffering from a vision-threatening AE will also be withdrawn from the study. Withdrawal from the study will result in exclusion of the data from analysis from those participants, unless adherence to the protocol and follow-up examinations were sufficient to allow inclusion in the analysis. The reason for withdrawal will be recorded in the CRF.

If the participant is withdrawn due to an AE, the investigator will make arrangements for follow-up visits or telephone calls until the AE has resolved or stabilized.

Sample size calculations showed the need for 78 participants per treating arm to find significant values. In case of a withdrawal, a replacement subject will be included. This could cause a delay to the end of the trial but is not relevant for the outcomes. All subjects withdrawn from this study will return to normal consultation at their ophthalmologist of choice.

#### Safety reporting

##### Adverse event reporting period

The reporting period during which AEs must be reported is the period from enrollment to the end of the study period (24 months). All unresolved AEs must be followed by the trial monitor in contact with the chief investigator and principal investigators until the events are resolved, the patient is lost to follow-up, or the AE is otherwise explained. At the last scheduled study visit, the trial nurse will instruct each patient to report any subsequent event(s) that the patient, or the patient’s personal physician, believes might reasonably be related to prior study treatment. Such events should be reported to the (previous) treating ophthalmologist at the department of ophthalmology of the trial site after the trial has ended. Patients who withdraw early from the study will be contacted by trial staff 30 days after their last visit, if the patient gives permission to do so, to ascertain whether any AEs have occurred.

##### *Definition of adverse events/reactions*

An *adverse event (AE)* or adverse experience includes any untoward medical occurrence in a patient or clinical investigation participant who has been administered a medicinal product, and this event does not necessarily have to have a causal relationship with this treatment (the study medication).

An AE can therefore be any unfavorable and unintended sign (including an abnormal laboratory finding), symptom or disease temporally associated with the use of the study medication, whether or not the event is considered related to the study medication.

In the case of an *adverse reaction (AR)*, a causal relationship between a study medication and an AE is at least a reasonable possibility (that is, the relationship cannot be ruled out).

All cases judged by either the reporting medically qualified professional or the sponsor as having a reasonable suspected causal relationship to the study medication qualify as adverse reactions.

These include all untoward and unintended responses to a medicinal product related to any dose.

ARs could include, for instance, nausea, hypertension, lower back pain*,* headache*,* arthralgia, intravenous injection site reactions (in the case of PDT), cough, pharyngitis, pneumonia, fever and flu-like symptoms, hypersensitivity and allergy reactions, vaso-vagal reactions, atrial fibrillation, angina, and sunburn after sunlight exposure in the first two days after PDT treatment.

#### Procedures for recording adverse events/reactions

All AEs occurring during the study observed by the investigator or reported by the participant, whether or not attributed to study medication, will be recorded on the CRF. The following information will be recorded: description, date of onset and end date, severity, assessment of relatedness to study medication, other suspect drug or device and action taken. Follow-up information should be provided as necessary. AEs considered related to the study medication, as judged by a medically qualified investigator or the sponsor, will be followed until resolution or until the event is considered stable. AEs considered related to the study procedure, as judged by a qualified investigator or the sponsor, will be followed until resolution or until the event is considered stable. All related AEs that result in a participant’s withdrawal from the study or are present at the end of the study, should be followed up if the patient gives consent to do so, until a satisfactory resolution occurs.

It will be left to the investigator’s clinical judgment whether or not an AE is of sufficient severity to require the participant’s removal from the trial. A participant may also voluntarily withdraw from treatment due to what he or she perceives as an intolerable AE. If either of these occurs, the participant must undergo an end-of-study assessment and be given appropriate care under medical supervision until symptoms cease or the condition becomes stable.

The relationship of AEs to the study medication will be assessed by a medically qualified investigator, and if necessary, discussed with the chief investigator. Any pregnancy occurring during the clinical study and the outcome of the pregnancy fathered by trial participants, should be recorded and followed up for congenital abnormality or birth defect.

#### Definitions of serious adverse events/reactions

A *serious adverse event (SAE)* is any untoward medical occurrence that at any dose accomplishes the following:

1. Results in death,
2. Is life-threatening, note: the term "life-threatening" in the definition of "serious" refers to an event in which the participant was at risk of death at the time of the event; it does not refer to an event that hypothetically might have caused death if it were more severe.
3. Requires inpatient hospitalization or prolongation of existing hospitalization.
4. Results in persistent or significant disability/incapacity, or is a congenital anomaly/birth defect.
5. Other important medical events. Note: other events that may not result in death, are not life threatening, or do not require hospitalization, may be considered a SAE when, based upon appropriate medical judgement, the event may jeopardize the patient and may require medical or surgical intervention to prevent one of the outcomes listed above. In this trial, these events are mainly vision-related.

##### Vision-threatening adverse events/reactions

An AE is considered to be vision-threatening and is a reportable SAE if it meets one or more of the following criteria:

1. It caused a decrease in visual acuity of >30 letters (compared with the last assessment of visual acuity prior to the most recent treatment) within the follow-up period of 7 to 8 months.
2. It required surgical intervention.
3. In the investigator’s opinion, it may require medical intervention to prevent permanent loss of vision. Causes for such vision-related AEs could include for instance: RPE tears, subretinal hemorrhage, choroidal neovascularization.

A *serious adverse reaction (SAR)* is an adverse event (expected or unexpected) that is both serious and, in the opinion of the reporting investigator, believed with reasonable probability to be due to one of the study treatments, based on the information provided.

A *suspected unexpected serious adverse reaction (SUSAR)* is a serious adverse reaction, the nature or severity of which is not consistent with the applicable product information (for example, the Investigator’s brochure for an unapproved investigational product or summary of product characteristics for an approved product).

#### Reporting procedures for serious adverse events

The data monitoring committee (DMC) will undertake a review of all SAEs for the study. The DMC may hold electronic meetings. The DMC will meet at intervals and consider the following:

1. The occurrence and nature of the adverse events.
2. Whether additional information on the adverse events is required.
3. Taking appropriate action where necessary to halt trials.
4. Act/advise on incidents occurring between meetings that require rapid assessment (for example, SUSARs).

All SAEs will be reported to the DMC within one working day of discovery or notification of the event. All SAE information will be recorded on an SAE form, which will be sent electronically to members of the DMC. Additional information received for a case (follow-up or corrections to the original case) will be detailed on a new SAE form. After receiving the SAE report within one working day, the medical monitor will review possible SAEs weekly, and the DMC has a meeting every three months to review the SAEs, if present. The chief investigator will also report all SUSARs to the competent authorities (TOL/CCMO in the Netherlands, Bfarm in Germany, and ANSM (Agence Nationale de Sécurité du Médicament et des Produits de Santé) in France, the ethics committees concerned, and the host NHS trust in the United Kingdom. Fatal or life-threatening SUSARs will be reported within 7 days and all other SUSARs within 15 days. The Chief Investigator will also inform all investigators concerned of relevant information about SUSARs that could adversely affect the safety of participants.

#### Trial safety group/data monitoring committee

The DMC will conduct a review of all SAEs for the study reported every 3 months and cumulatively, if present. The aims of this review will include the following:

1. To pick up any trends, such as increases in unexpected/expected events, and take appropriate action.
2. To seek additional advice or information from investigators where required.
3. To evaluate the risk of the trial continuing and take appropriate action where necessary.
4. To act or advise, through the chairman or other medical monitors, on incidents occurring between meetings that require rapid assessment.

This committee, which is based in the coordinating center in Nijmegen, will receive SAEs within one working day, and will analyze the available safety data and study data in a meeting every 3 months, if present.

### Description of statistical methods

#### Univariate analysis

##### *Analysis for the primary endpoint*

The purpose of this study is to identify the difference between the efficacy of the two treatment modalities based on the anatomical effect on OCT scan (absence of SRF versus still SRF visible). As we expect one treatment to be superior to the other, this study is designed to be a superiority study. Statistical analysis on the primary parameter will be performed by analyzing the relative risk by using a cross-table comparing evaluation at 6 to 8 weeks after treatment with baseline.

#### Multivariate analysis

##### Analysis for the secondary endpoints

For the secondary endpoints, the following analyses will be performed:

1. Anatomic results based on OCT scan (absence of SRF versus persistent SRF) at evaluation point 1 will be compared to the baseline.
2. Number of subsequent treatments needed in each treatment arm.
3. Mean change from baseline in ETDRS BCVA in the study eye at 6 to 8 weeks after treatment and at the end evaluation will be compared between the two treatment modalities.
4. Mean change from evaluation point 1 in ETDRS BCVA in the study eye at final evaluation among those with subsequent and those without subsequent treatment will be compared between the two treatment modalities
5. Mean change from baseline in retinal sensitivity in the study eye to 6 to 8 weeks after treatment will be compared between the two treatment modalities.
6. Mean change from baseline in the NEI-VFQ-25 questionnaire to 6 to 8 weeks after treatment will be compared among the two treatment modalities.

The first two analyses will be performed by the use of a cross-table. Furthermore, the continuous secondary variables will be analyzed using an ANCOVA model with baseline as and treatment as factor. For categorical secondary endpoints, a chi-square test will be performed. In addition, a logistic model with baseline as covariate and treatment as factor will be performed. The change from baseline in the NEI-VFQ-25 questionnaire results will be summarized descriptively.

#### Interim analysis

A formal interim analysis will be performed when 78 participants received the first evaluation at 6 to 8 weeks after treatment. Statistical analysis on the primary parameter will be performed by analyzing the relative risk by using a cross-table comparing evaluation at 6 to 8 weeks after treatment with baseline.

#### Number of participants

*Justification of sample size: total number of patients is 156*

For HSML, an anatomic success rate of approximately 50 %, defined as no SRF on OCT, after 6 to 8 weeks may be estimated based on retrospective studies [48, 56]. For half-dose PDT, approximately 80 % anatomic success may be estimated [18, 26–35]. Taking such a difference of 30 % in treatment success into account, a power calculation indicates that one would need 40 patients per treatment arm (power: 80 %, α: 0.05). However, when we also correct for factors such as positive publication bias and our own empiric treatment experience, we expect to find a difference in treatment success rate of approximately 22 % in favor of half-dose PDT. If we take one interim analysis into account, according to the O’Brien-Fleming method, 78 patients per treatment arm would be required (power: 80 %, α: 0.05).

*Distribution of subjects per site*

**Table:** 

	Netherlands (Nijmegen/Leiden)	Oxford (UK)/Cologne (Germany)/Paris (France)
Group 1*	50 subjects	28 subjects
Group 2*	50 subjects	28 subjects

*group 1 (78 patients): half-dose PDT with Visudyne™

*group 2 (78 patients): HSML

#### The level of statistical significance

For the primary endpoint, the overall null hypothesis is as follows:

H_0_ : probabilities of success are the same in both treatment arms (π_1_ = π_2_)

*versus*

H_1_ : probabilities of success are different in the treatment arms (π_1_ ≠ π_2_)

For statistical testing, the significance level will be 0.05 unless specified otherwise.

### Criteria for the termination of the trial

The DMC will perform a review of the study data every 3 months. The study may be terminated prematurely on the recommendation of the DMC. Reasons for premature termination of the trial may include the following:

1. Early solid statistical evidence that the investigational medicinal product (IMP) is significantly better than the comparator.
2. Early evidence that one or both treatments are harmful.

### Ethics

#### Declaration of Helsinki

The Chief Investigator will ensure that this study is conducted in accordance with the principles of the 2008 Declaration of Helsinki.

### ICH guidelines for good clinical practice

The investigator will ensure that this study is conducted in full conformity with relevant regulations and with the ICH guidelines for good clinical practice (CPMP/ICH/135/95) July 1996.

### Approvals

The protocol, informed consent form, participant information sheet, and any proposed advertising material was submitted to and received written approval from the appropriate research ethics committees (REC) (see list below), regulatory authorities, and host institution(s). This trial has been internationally registered at Clinicaltrials.gov (NCT01797861; ClinicalTrials.gov).

The research ethical committees that have approved the study protocol at the different sites are listed below:

1. The Commissie Mensgebonden Onderzoek (CMO) Regio Arnhem-Nijmegen approved the protocol for the following locations:
  Radboud University Medical Center, Nijmegen, the Netherlands, and
  Leiden University Medical Center, Leiden, the Netherlands.
2. The Ethik Kommission Universität zu Köln approved the protocol for the following location:
  University Hospital Cologne, Cologne, Germany.
3. The Health Research Authority National Research Ethics Service (NRES) Committee South Central – Oxford A approved the protocol for the following location:
  Oxford Eye Hospital, Oxford, United Kingdom
4. The Comité de Protection des Personnes Ile de France V approved the protocol for the following location:
  University Paris Est Creteil, Paris, France

## Discussion

To establish the optimal treatment for cCSC, an eye disease associated with potentially severe visual disability, a multicenter prospective randomized controlled trial is mandatory but currently lacking. The proposed study is the first multicenter, prospective, randomized controlled trial that compares half-dose PDT with HSML treatment with regard to their ability to reduce SRF accumulation in cCSC, and their ability to improve the quality of vision. We have chosen to adopt half-dose PDT treatment instead of half-fluency PDT treatment. A reduction of the dose of verteporfin, rather than the fluency of the laser treatment, appears preferable because a dose reduction may reduce possible systemic side effects of PDT treatment, such as lower back pain and photosensitivity of the skin in the first days after treatment [57], while having an efficacy comparable to half-fluency PDT [58, 59].

HSML treatment has been chosen as the treatment of choice in the control arm for a number of reasons. First, sham (no treatment) was studied by Chan and co-workers in the acute form of CSC, who showed a large difference in anatomic and functional outcome (complete resolution of SRF) between the half-dose PDT and placebo group [60]. As it is well established that prolonged leakage of SRF under the macula due to cCSC may lead to permanent visual loss, it is not desirable to include comparison with sham in our study [8–10, 20, 25, 60–65]. HSML treatment of CSC has been shown to be effective and safe in 41-58 % of patients in smaller, retrospective studies [18, 48, 56]. The safety and efficacy of HSML treatment has also been reported in various other retinal diseases [49]. In contrast, it has been shown that conventional laser treatment of focal leakage point on fluorescein angiography in CSC does not result in a better visual outcome [3, 41]. Also, conventional laser treatment in CSC has a higher risk of complications than HSML and half-dose PDT, including vision loss, scotoma, decreased color vision, decreased contrast sensitivity, and choroidal neovascularization [2, 41, 66]. The 810 nm HSML laser modality was chosen instead of the 577 nm wavelength because at the time of the start of the study, relatively little had been published on the safety and efficacy of 577 nm HSML, and experimental studies between 810 nm and 532 nm micropulse laser strategies have not shown obvious differences in histological effect [67].

## Trial status

The first participant was recruited on 18 December 2013, and the estimated study completion date is June 2017.

## Abbreviations

AE: adverse event
AR: adverse reaction
BCVA: best-corrected visual acuity
CI: Chief Investigator
CRC: Central Reading Center
CRF: case report form
CSC: central serous chorioretinopathy
cCSC: chronic central serous chorioretinopathy
DMC: data monitoring committee
ETDRS: Early Treatment of Diabetic Retinopathy Study
GP: General Practitioner
HSML: high-density subthreshold micropulse laser
ICG: indocyanine green
ICH-GCP: International Convention of Helsinki - Good Clinical Practice
NEI-VFQ-25: National Eye Institute Visual Function Questionnaire 25
OCT: optical coherence tomography
PDT: photodynamic therapy
PI: Principal Investigator
R&D: Research and Development
REC: research ethics committees
RPE: retinal pigment epithelium
SAE: serious adverse event
SAR: serious adverse reaction
SOP: standard operating procedure
SRF: subretinal fluid
SUSAR: suspected unexpected serious adverse events
TMF: trial master file
